# Tetracycline hypersensitivity of an *ezrA* mutant links GalE and TseB (YpmB) to cell division

**DOI:** 10.3389/fmicb.2015.00346

**Published:** 2015-04-22

**Authors:** Pamela Gamba, Eva Rietkötter, Richard A. Daniel, Leendert W. Hamoen

**Affiliations:** ^1^Centre for Bacterial Cell Biology, Institute for Cell and Molecular Biosciences, Newcastle UniversityNewcastle upon Tyne, UK; ^2^Bacterial Cell Biology, Swammerdam Institute for Life Sciences, University of AmsterdamAmsterdam, Netherlands

**Keywords:** FtsZ, EzrA, tetracycline, FtsL, GalE, *Bacillus subtilis*

## Abstract

Cell division in bacteria is initiated by the polymerization of FtsZ into a ring-like structure at midcell that functions as a scaffold for the other cell division proteins. In *Bacillus subtilis*, the conserved cell division protein EzrA is involved in modulation of Z-ring formation and coordination of septal peptidoglycan synthesis. Here, we show that an *ezrA* mutant is hypersensitive to tetracycline, even when the tetracycline efflux pump TetA is present. This effect is not related to the protein translation inhibiting activity of tetracycline. Overexpression of FtsL suppresses this phenotype, which appears to be related to the intrinsic low FtsL levels in an *ezrA* mutant background. A transposon screen indicated that the tetracycline effect can also be suppressed by overproduction of the cell division protein ZapA. In addition, tetracycline sensitivity could be suppressed by transposon insertions in *galE* and the unknown gene *ypmB*, which was renamed *tseB* (*t*etracycline sensitivity *s*uppressor of *e*zrA). GalE is an epimerase using UDP-glucose and UDP-N-acetylglucosamine as substrate. Deletion of this protein bypasses the synthetic lethality of *zapA ezrA* and *sepF ezrA* double mutations, indicating that GalE influences cell division. The transmembrane protein TseB contains an extracytoplasmic peptidase domain, and a GFP fusion shows that the protein is enriched at cell division sites. A *tseB* deletion causes a shorter cell phenotype, indicating that TseB plays a role in cell division. Why a deletion of *ezrA* renders *B. subtilis* cells hypersensitive for tetracycline remains unclear. We speculate that this phenomenon is related to the tendency of tetracycline analogs to accumulate into the lipid bilayer, which may destabilize certain membrane proteins.

## Introduction

Division of a bacterial cell involves the coordinated action of several proteins that localize at midcell and assemble in a multiprotein complex known as the divisome. The most crucial component of the division machinery is FtsZ, a structural homolog of eukaryotic tubulin (Lowe and Amos, 1998), which polymerizes into a ring-like structure at midcell when cell division is initiated (Bi and Lutkenhaus, 1991; Peters et al., 2007). In *B. subtilis*, the Z-ring is tethered to the membrane by FtsA (Wang et al., 1997; Ma and Margolin, 1999) and SepF (Duman et al., 2013), and functions as a scaffold for all other division proteins (for review, see Adams and Errington, 2009). Bundling of FtsZ protofilaments is stimulated by both SepF and the conserved protein ZapA (Gueiros-Filho and Losick, 2002; Singh et al., 2008; Gundogdu et al., 2011; Pacheco-Gomez et al., 2013). Another conserved early cell division protein that binds to the Z-ring is EzrA, which will be discussed below. After the Z-ring has formed, the late cell division proteins are recruited (Gamba et al., 2009). The transmembrane proteins PBP 2B, FtsL, DivIB, and DivIC are interdependent for their recruitment to the Z-ring. PBP 2B is the transpeptidase that introduces cross-links into septal peptidoglycan (Daniel et al., 1996). The exact function of FtsL, DivIB, and DivIC is unclear. FtsL is efficiently degraded by the regulatory protease RasP through intramembrane proteolysis (Bramkamp et al., 2006), and presumably plays a regulatory role because of its marked instability (Daniel et al., 2006). DivIC is also an unstable protein (Robson et al., 2002). DivIB might have a role in the regulation of FtsL and DivIC stability (Daniel et al., 2006).

Several proteins modulate the assembly of the Z-ring in space and time. In *Bacillus subtilis*, the Min proteins prevent cell division at newly formed cell poles by inhibiting FtsZ bundling (Dajkovic et al., 2008; Gregory et al., 2008), and by promoting disassembly of the divisome after division is completed (van Baarle and Bramkamp, 2010). The nucleoid occlusion protein Noc binds to specific DNA sequences and prevents Z-ring assembly over the nucleoid, thereby coordinating cell division with DNA segregation (Wu and Errington, 2004; Wu et al., 2009; Adams et al., 2015). Cell division also responds to the metabolic status of the cell. The glucosyltransferase UgtP, involved in the synthesis of lipoteichoic acids, has been shown to accumulate at septa and to inhibit cell division in a growth-rate dependent manner (Weart et al., 2007; Chien et al., 2012). Recently, a link to central carbon metabolism has been established with the discovery that pyruvate levels can affect Z-ring formation (Monahan et al., 2014).

The early cell division protein EzrA is conserved in low G+C Gram-positive bacteria. The protein is anchored to the cell membrane by an N-terminal transmembrane domain and has a large cytoplasmic C-terminal domain that binds to FtsZ (Levin et al., 1999; Haeusser et al., 2004). Initially, it was assumed that EzrA negatively regulates Z-ring formation since an *ezrA* mutant shows an increased frequency of Z-rings in fast growth rate conditions, and purified EzrA inhibits bundling of FtsZ protofilaments (Haeusser et al., 2004; Chung et al., 2007; Singh et al., 2007). However, the function of EzrA is more complicated. Cells lacking EzrA are significantly longer than wild-type cells because of a delay in constriction (Levin et al., 1999; Kawai and Ogasawara, 2006), and deletion of the positive Z-ring regulators *zapA* or *sepF* in an *ezrA* background causes a severe block in cell division (Gueiros-Filho and Losick, 2002; Hamoen et al., 2006). A recent crystallographic study suggested that EzrA forms large semi-circular structures that can hook FtsZ filaments to the cell membrane. The large curved EzrA structures show some homology to the spectrin proteins which connect actin filaments in eukaryotes (Cleverley et al., 2014). Another activity of EzrA is the recruitment of the major transglycosylase/transpeptidase PBP 1 from the lateral wall to the division site (Claessen et al., 2008; Tavares et al., 2008).

Here we describe a peculiar phenotype of an *ezrA* mutant, the hypersensitivity to the antibiotic tetracycline. Detailed analysis of this phenomenon revealed that this sensitivity is not related to the classical inhibitory effect of tetracycline on protein translation. We show that overexpression of FtsL can suppress the tetracycline effect, and low levels of this key cell division regulator might be the reason for the phenotype. Using an extensive transposon screen we identified two new genes, *galE* and *ypmB*, which suppress the tetracycline sensitivity of an *ezrA* mutant when deleted. Interestingly, the absence of the UDP-galactose epimerase GalE restores also the lethal cell division defects of a *ezrA sepF* or *ezrA zapA* double mutant. Since a transposon insertion in the unknown *ypmB* gene suppresses the tetracycline induced defects of an *ezrA* mutant, the gene was renamed *tseB* (*t*etracycline sensitivity *s*uppressor of *e*zrA). TseB is a membrane protein with an extracellular protease domain, and is enriched at cell division sites. The absence of this protein causes a short cell phenotype, further suggesting a role in cell division.

## Materials and methods

### Bacterial strains and growth conditions

Strains and plasmids used in this study are listed in Table 1. *B. subtilis* strains were grown at 30°C or 37°C in Antibiotic medium no. 3 (PAB, Difco, or Oxoid), LB or competence medium (CM) (Hamoen et al., 2002). Agar (Bacteriological agar no. 1, Oxoid) was added to a final concentration of 1.5% to prepare solid media. When required, media were supplemented with 10 μg/ml tetracycline (unless stated otherwise), 5 mM MgSO_4_, 22.5 μM EDTA, 22.5 μM phenanthroline, or 0.5 μg/ml anhydrotetracycline. If needed, xylose and IPTG were used as inducers at concentrations of 0.5–2%, and 1 mM, respectively. Selection of transformants was performed on nutrient agar (Oxoid), supplemented when required with 10 μg/ml tetracycline, 5 μg/ml chloramphenicol, 50 μg/ml spectinomycin, 5 μg/ml kanamycin or 0.5 μg/ml erythromycin with 25 μg/ml lincomycin. *E. coli* strains were grown in LB at 37°C and used as cloning intermediates.

**Table 1:** **Strains and plasmids used in this study**.

**Strain**	**Relevant features or genotype**	**Construction, source or reference**
***B. subtilis***		
168	*trpC2*	Laboratory stock
BSB1	*trp+*	Nicolas et al., 2012
1356	*zapA-yshB::tet*	Feucht and Errington, 2005
2020	*amyE*::*(*P*_xyl_-gfpmut1-ftsZ, spc)*	J. Sievers (unpublished)
3362	*ezrA*::*tet*	Hamoen et al., 2006
3828	*ftsL*::*pSG441 aphA-3 P_spac_-pbpB, amyE*::*cat P_xyl_-Δ30-ftsL*	Bramkamp et al., 2006
4077	*ylmBC*::*(erm P_spac_-ylmD), ezrA*::*tet*	Hamoen et al., 2006
814	*ΔftsL-Pspac-pbpB kan, amyE*::*Pxyl-HA-ftsL cat*	Daniel and Errington, 2000
BG239	*thr-5, tet-4*	Wei and Bechhofer, 2002
KS273	*aprE*::*Pspac-zapA spc*	Surdova et al., 2013
LH28	*ezrA*::*cat*	L. Hamoen (unpublished)
SG82	*lacA*::*tet*	Laboratory stock
YK012	CRK6000 *ezrA*::*spc*	Kawai and Ogasawara, 2006
YK204	CRK6000 *sepF*::*spc*	Ishikawa et al., 2006
PG49	*ezrA*::*spc*	YK012 DNA → 168
PG100	*lacA*::*tet, ezra*::*cat*	LH28 DNA → SG82
PG112	*tet-4*	5kb *rpsJ* region from BG239 → 168
PG113	*tet-4, ezrA*::*spc*	5kb *rpsJ* region from BG239 → PG49
PG116	*tet-4, ezrA*::*cat*	5kb *rpsJ* region from BG239 → LH28
PG121	*ezrA*::*tet*, *tseB*:*TnYLB-1 kan*	pMarB integration into 3362
PG126	*ezrA*::*tet*, *zapA-TnYLB-1- yshB kan*	pMarB integration into 3362
PG129	*ezrA*::*tet*, *galE*:*TnYLB-1 kan*	pMarB integration into 3362
PG135	*tseB*:*TnYLB-1 kan*	PG121 DNA → 168
PG140	*zapA-TnYLB-1- yshB kan*	PG126 DNA → 168
PG143	*galE*:*TnYLB-1 kan*	PG129 DNA → 168
PG149	*aprE*::*P_spac_-zapA, spc*	KS273 DNA → 168
PG158	*sepF*::*spc*	YK204 DNA → 168
PG160	*ezrA*::*tet, amyE*::*P_xyl_-Δ30ftsL-cat*	3828 DNA → 3362
PG162	*ezrA*::*cat, aprE*::*P_spac_-zapA spc*	PG149 DNA → LH28
PG164	*ezrA*::*cat, zapA-yshB*::*tet, P_spac_-zapA*	1356 DNA → PG162
PG209	*ezrA*::*tet, amyE*::*P_xyl_-gfp-ftsZ spc*	2020 DNA → 3362
PG234	*galE*::*kan*	This work
PG235	*tseB*::*kan*	This work
PG238	*ezrA*::*tet, galE*::*kan*	PG234 DNA → 3362
PG239	*ezrA*::*tet, tseB*::*kan*	PG235 DNA → 3362
PG251	*galE*::*spc*	This work
PG252	*tseB*::*spc*	This work
PG294	*ezrA*::*tet, galE*::*kan, sepF*::*spc*	YK204 DNA → PG238
PG296	*galE*::*kan, ylmBC*::*(erm P_spac_-ylmD), ezrA*::*tet*	4077 DNA → PG238
PG305	*ΔftsL-P_spac_-pbpB kan, amyE*::*P_xyl_-HA-ftsL cat, ezrA*::*tet*	3362 DNA → 814
PG307	*ezrA*::*cat, zapA-yshB*::*tet, aprE*::*P_spac_-zapA spc, galE*::*kan*	PG234 DNA → PG164
PG325	*aprE*::*P_spac_-tseB spc*	pPG16 → 168
PG327	*aprE*::*P_spac_-galE spc*	pPG18 → 168
PG330	*tseB*::*kan, aprE*::*P_spac_-tseB spc*	PG325 DNA → PG235
PG332	*ezrA*::*tet, galE*::*kan, aprE*::*P_spac_-galE spc*	PG327 DNA → PG239
PG333	*ezrA*::*tet, tseB*::*kan, aprE*::*P_spac_-tseB spc*	PG325 DNA → PG239
PG718	*trp+, amyE*::*P_xyl_-gfp-tseB spc*	pPG6(mGFP) integration into BSB1
PG742	*ΔftsL-Pspac-pbpB kan, amyE*::*Pxyl-HA-ftsL cat, lacA*::*tet*	SG82 DNA → 814
***E. coli***		
DH5α	*F*^−^, *φ80lacZΔM15*, *Δ(lacZYAargF)U*196, *recA1*, *endA1*, *hsdR17*, (*r*_*K*−,_ *m*_*K*+_), *phoA*, *supE44*, λ^−^, *thi*^−^1, *gyrA96*, *relA1*	Invitrogen
**Plasmid**	**Relevant features or genotype**	**Construction, source or references**
pAPNC213	*bla aprE' spc lacI P_spac_'aprE*	Morimoto et al., 2002
pMarB	*bla erm P_ctc_ Himar1 kan* (TnYLB-1)	Le Breton et al., 2006
pSG1729	*bla amyE3' spc P_xyl_-gfpmut1' amyE5'*	Lewis and Marston, 1999
pHT21	*kan*	Trieu-Cuot and Courvalin, 1983
pLOSS*	*spc*	Claessen et al., 2008
pPG6(mGFP)	*bla amyE3' spc P_xyl_tseB-mgfpm1' amyE5'*	This work
pPG16	*bla aprE' spc lacI P_spac_ tseB'aprE*	This work
pPG18	*bla aprE' spc lacI P_spac_ galE'aprE*	This work

*Unless stated otherwise, all strains were made in the 168 wild type background. Genes responsible for resistance to antibiotics are abbreviated as follows: bla, ampicillin; cat, chloramphenicol; erm, erythromycin; kan, kanamycin; spc, spectinomycin; tet, tetracycline*.

### Growth assays on agar plates

Frozen stocks were streaked out to single colonies on nutrient agar plates supplemented as required and grown overnight at 37°C. To ensure an even distribution of cells on all the plates that had to be compared, fresh single colonies were picked and spread onto a short streak on a new nutrient agar plate. This primary streak was then crossed with a new sterile loop that was used to transfer the inoculum on a new plate and isolate single colonies. Then, for each of the other agar plates that had to be compared within the same experiment, the same procedure was repeated with a new sterile loop by crossing the primary streak on a different (yet adjacent) point.

### Plasmid and strain construction

Molecular cloning, PCRs, and *E. coli* transformations were carried out by standard techniques. Oligonucleotides used in this study are listed in Table 2. To construct plasmid pPG6(mGFP), a fragment of 504 bp containing the *tseB* coding sequence was amplified from 168 chromosomal DNA with oligonucleotides PG77 and PG79, carrying the *Hind*III and *Xho*I restriction sites, respectively. The insert was then cloned into an equally cut pSG1729, resulting in plasmid pPG6. Plasmid pPG6 was then used for a quick change mutagenesis reaction with oligonucleotides HS410 and HS411 in order to introduce the A206K mutation in the GFP coding sequence to reduce protein dimerization. The resulting plasmid was verified by sequencing and named pPG6(mGFP).

**Table 2:** **Oligonucleotides used in this study**.

**Name**	**Restriction site**	**Sequence (5′–3′)**
HS410		CCTGTCCACACAATCTAAACTTTCGAAAGATCCC
HS411		GGGATCTTTCGAAAGTTTAGATTGTGTGGACAGG
Km3	*Bam*HI	GGG**GGATCC**AAGACGAAGAGGATGAAG
Km4	*Eco*RI	CCC**GAATTC**AGAGTATGGACAGTTGCG
oIPCR1		GCTTGTAAATTCTATCATAATTG
oIPCR2		AGGGAATCATTTGAAGGTTGG
oIPCR3		GCATTTAATACTAGCGACGCC
PG57		TGATGGTGCTCCAGAAGAAC
PG58		ACAGAACCACGAACTGTAGG
PG70	*Eco*RI	GCC**GAATTC**TTATTCCGCACTCTTATACCCATT
PG77	*Hind*III	CGG**AAGCTT**TTAAGGCGTGATATTTTTGAGAA
PG79	*Xho*I	CGG**CTCGAG**ATGAGAAAAAAAGCATTAATATTTACCG
PG103	*Bam*HI	GAC**GGATCC**CTTCTCACCTACGTACGATA
PG120		TACCTTCCTGCAGCTGATTC
PG121		GAGCAGCTTACTGGAATCTC
PG122	*Hind*III	GATCAGT**AAGCTT**GACGAATTAGGGGGAGTTCAAG
PG128	*Bam*HI	GAA**GGATCC**CCTAAAAAATGACCTGTTTT
PG129		CTCCGTTCCTCCACTTGATG
PG130	*Eco*RI	ATA**GAATTC**GAATGGAGGCCTTCTCAATT
PG131		ATGATGATCGCCCGCGAAAC
PG134	*Nco*I	GAGT**CCATGG**TCAGAGTATGGACAGTTGCG
PG135	*Nco*I	GATC**CCATGG**GACGAATTAGGGGGAGTTCAAG
PG146	*Bam*HI	CCGA**GGATCC**AGGATGTACTTAAACGCTAACG
PG149	*Hind*III	GCC**AAGCTT**CAAGAGGACGCTTTATTCTTC
PG152	*Eco*RI	CGTC**GAATTC**TTAAGGCGTGATATTTTTGAGAA
PG159	*Bam*HI	ACCT**GGATCC**TCGGCCTTGCGCTGGATGAAGA
PG161	*Bam*HI	GGCA**GGATCC**CTATTAATAAACGATTAAACTTC
Spc-pLoss-Rev	*Eco*RI	GCAGCC**GAATTC**CAAGAGGACGCTTTATTCTTC

*Recognition sites for restriction enzymes are indicated in bold*.

Plasmids pPG16 and pPG18 were derived from pAPNC213, which was digested with *Eco*RI and *Bam*HI, and ligated with PCR products digested with the same restriction enzymes. For plasmid pPG16, the *tseB* coding sequence, including the ribosome binding site, was amplified using oligonucleotides PG152 and PG159. For plasmid pPG18, the *galE* coding sequence and 70 bp of the upstream region was amplified with oligonucleotides PG70 and PG161.

Genes were deleted by replacing their coding sequences with antibiotic resistance cassettes. Approximately 3 kb upstream and downstream of the coding sequence of the gene of interest were amplified. For deletion of *galE*, oligonucleotides PG128-PG129 and PG130-PG131 were used. For *tseB* deletions, oligonucleotides PG120-PG135 and PG103-PG131 were used for a deletion with a *kan* cassette, while PG120-PG122 and PG103-PG121 were used to construct a deletion with a *spc* cassette. Relevant restriction sites were inserted into the primers. Ligation reactions were assembled with equimolar concentrations of each of the three PCR products, using about 1.5 μg of each 3 kb product in a total volume of 40 μl. Competent cells of *B. subtilis* were transformed with half of each ligation reaction. Transformants were selected on antibiotic plates and verified by PCR. Antibiotic resistance cassettes were amplified from plasmids: *kan* from pHT21 (oligonucleotides km3-km4 for *galE* and km3-PG134 for *tseB*), *spc* from pLOSS^*^ (oligonucleotides PG146 and spc-pLoss-Rev for *galE*, PG146-PG149 for *tseB*).

An N-terminal GFP fusion to *tseB* was constructed by transforming pPG6(mGFP) plasmid into strain BSB1, generating strain PG718. The integration was obtained by a double crossover recombination event between the *amyE* regions located on the plasmid and the chromosomal *amyE* gene of strain 168. Transformants were selected on nutrient agar plates containing spectinomycin. Correct integration into the *amyE* gene was tested and confirmed by lack of amylase activity upon growth on plates containing 1% starch.

### Microscopic imaging

Samples were mounted on microscope slides coated with a thin layer of 1.2% agarose. Images were acquired with a Zeiss Axiovert 200 M or a Zeiss Axiovert 135 microscope coupled to a Sony Cool-Snap HQ cooled CCD camera (Roper Scientific), and using Metamorph imaging software (Universal Imaging). For membrane staining, cells were mounted on slides coated with 1% agarose supplemented with the membrane dye Nile Red (0.1 μg/ml, Molecular Probes) or by mixing 9 μl of cells with 1 μl of Nile Red solution (12.5 mg/ml) before spotting the sample on the agarose slide. Alternatively, cells were mixed with the membrane dye FM5-95 (Invitrogen), at a final concentration of 0.4 μg/ml. Nucleoids were stained by adding DAPI (0.02 μg/ml, Sigma) to the agarose slide. Images were analyzed and prepared for publication with ImageJ (http://rsb.info.nih.gov/ij/). For time-lapse microscopy, strain PG718 was grown in CM supplemented with 0.5% xylose at 30°C until cells reached exponential phase, and subsequently mounted onto a thin semisolid matrix made of CM supplemented with 0.5% xylose and 1.5% low-melting point agarose on a microscope slide. Slides were incubated in a temperature-controlled chamber (30°C) on a Deltavision RT automated microscope (Applied Precision). Phase contrast and GFP images were taken every 10 min.

### Screen for tetracycline-insensitive suppressor mutants

Random transposon mutagenesis of strain 3362 (*ezrA*::*tet*) was carried out using the mariner transposable element TnYLB-1 as described (Le Breton et al., 2006). Plasmid pMarB was transformed into strain 3362 at 30°C. Individual colonies carrying the complete transposon plasmid were picked and grown in LB at 37°C for 8 h. Aliquots were frozen and stored at −80°C. Serial dilutions of each culture were plated on nutrient agar plates containing kanamycin or erythromycin and incubated at 50°C overnight to inhibit plasmid replication. The following morning, the clone with the highest ratio of kan^R^/erm^R^ colonies on plates was chosen. Appropriate dilutions of the selected clone were plated on nutrient agar plates and incubated at 50°C to construct a library of about 70,000 colonies. Cells were scraped off the plates, aliquoted, and frozen. About 75,000 clones of the library were plated on PAB plates supplemented with 10 μg ml^−1^ tetracycline and incubated at 37°C for 20 h. Individual colonies were picked and checked for integration of the transposon (kan^R^), loss of the plasmid (erm^S^), presence of the *ezrA* deletion (tet^R^), and checked under the microscope to see loss of the filamentous phenotype when streaked on PAB with tetracycline. Two rounds of backcrosses into strain 3362 were performed to confirm the linkage between transposon insertion and loss of tetracycline hypersensitivity. Finally, the site of transposon insertion was determined by performing an inverse PCR amplification on the chromosomal DNA which had been previously digested with TaqI and ligated. Finally, PCR reactions were sequenced and the results aligned with the *B. subtilis* published genome sequence. Oligonucleotides for inverse PCR and sequencing were oIPCR1, −2 and −3 respectively, as described (Le Breton et al., 2006).

### Cell length measurements

Cells were grown at 37°C in CM, LB, PAB, or PAB supplemented with 5 mM Mg^2+^. At mid-exponential phase for LB medium or early stationary phase for other media, cells were sampled and stained with Nile Red prior to microscopic examination. At least 100–150 cells were measured in each experiment and all experiments were replicated at least three times. The mean cell length was calculated for each experiment and then averaged over three replicates. Wild type cell length was set as 100% and relative cell length was calculated for all other strains.

### Western blotting

For the detection of HA-FtsL shown in **Figure 4**, cells were grown overnight at 30°C in PAB with 5 mM MgSO_4_, 0.5–2% xylose, 1 mM IPTG. Cultures were diluted to an O.D.600 of 0.1 in the same medium, grown for 2 h at 37°C and diluted again to 0.1 in warm medium. The exponentially growing cultures were incubated until an O.D.600 of 0.3 (**Figure 4A**) or 0.5 (**Figures 4B,C**) was reached. Cell pellets were resuspended in 100 μl of 1× NuPAGE LDS Sample Buffer (Invitrogen) with 5× Complete mini protease inhibitor (Roche) and broken by sonication. Relative protein concentrations were estimated by reading the A280 of all samples with a NanoDrop® ND-1000 spectrophotometer and equal amount of proteins were loaded on polyacrylamide gels. Proteins were transferred onto a PVDF membrane (GE Healthcare) by using either a wet or a semi-dry procedure and Western blotting was performed according to standard methods. In this study, a 1:10,000 dilution of rabbit polyclonal anti-FtsZ serum (laboratory stock), 1:10,000 dilution of rabbit polyclonal anti-PBP2B serum (laboratory stock), and a 1:1000 dilution of mouse monoclonal 12CA5 anti-HA antibody (Ivanov and Nasmyth, 2005) were used. Secondary antiserums, anti-rabbit-horseradish-peroxidase, and anti-mouse-horseradish-peroxidase (Sigma), were used at a dilution of 1:10,000.

For the immunodetection of ZapA shown in Figure S1, strains were grown at 37°C in PAB medium and samples were collected and flash frozen at O.D.600 ~0.3. Cell pellets were resuspended in lysis buffer (100 mM Tris-Cl pH 7.5, 2 mM EDTA, supplemented with Roche Complete mini protease inhibitor) containing 5 μg/ml lysozyme, incubated 10 min at 37°C and then sonicated. Cell debris were removed by centrifugation. Relative protein concentrations were estimated with a Bio-Rad protein assay and equal amount of proteins were loaded on NuPAGE 4–12% Bis-Tris gradient gels which were run in MES buffer (Life Technologies). Proteins were transferred onto a Hybond-P PVDF membrane (GE Healthcare) by using a wet procedure and western blotting was performed according to standard methods. A 1:2000 dilution of rabbit polyclonal anti-ZapA serum was used. Anti-rabbit horseradish peroxidase-linked antiserum (Sigma) was used as secondary antibody at a dilution of 1:10,000. Protein bands were detected using an ImageQuant LAS 4000 mini digital imaging system (GE Healthcare).

## Results

### Tetracycline hypersensitivity of an *ezrA* mutant

**Figure 1:**
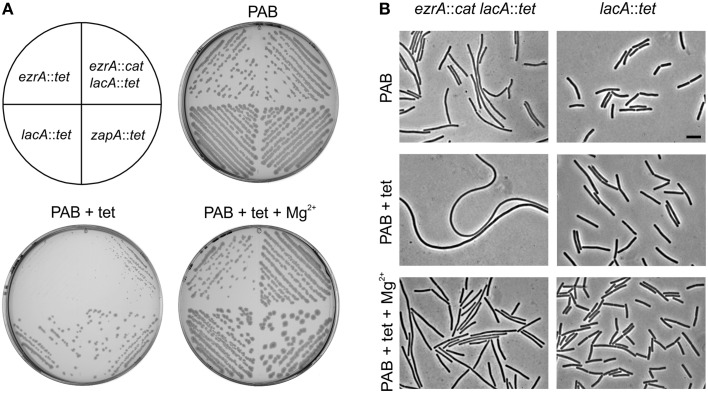
**Effect of tetracycline on *ezrA* mutants. (A)** Growth of *ezrA*::*tet* (3362), *lacA::tet* (SG82), *ezrA*::*cat lacA*::*tet* (PG100), and *zapA-yshB*::*tet* (1356) strains on PAB plates with and without 10 μg/ml tetracycline (tet) and 5 mM MgSO_4_ (Mg^2+^). **(B)** Phase contrast images of cells taken from the PAB plates in **(A)**. Scale bar 5 μm.

A *B. subtilis ezrA* mutant forms normal colonies on plate. When we transformed an *ezrA* deletion into other *B. subtilis* backgrounds, sometimes very small colonies were obtained that contained filamentous cells. However, this result was not always reproducible. Eventually, it emerged that this filamentous phenotype was caused by insertion of the tetracycline resistance cassette *tetL* in *ezrA*, in combination with selection of transformants on selective PAB plates. Without tetracycline, an *ezrA* mutant forms normal colonies on PAB plates (Figure 1A). Despite the presence of a functional resistance cassette, the addition of tetracycline (10 μg/ml) results in very small colonies containing highly filamentous cells, (Figures 1A,B). Addition of 5 mM MgSO_4_ to PAB plates with tetracycline restored normal growth and abolished filamentation (Figures 1A,B). When the *tetL* cassette was located at another locus (*lacA*), in an otherwise wild-type background, no effect on cell length or colony size was observed. Subsequent introduction of a different *ezrA* deletion (*ezrA*::*cat*) into this background resulted again in small colonies and strong filamentation on PAB plates with tetracycline (Figures 1A,B). For unknown reasons, we did not observe this filamentation phenotype in liquid PAB medium. Finally, hypersensitivity became apparent also on nutrient agar plates, but only when increased levels of tetracycline were used (≥ 30 μg/ml).

To examine whether the effect of tetracycline was specific for an *ezrA* mutant, several division mutants were tested that carried a *tetL* cassette. No significant effect on colony size or cell length was observed when a *zapA*, *sepF*, *noc*, or *gpsB* mutant was grown on PAB plates with tetracycline (Figure 1A and data not shown), indicating that the effect is specific for *ezrA*.

### Tetracycline effect is not related to protein translation inhibition

PAB medium contains relative low concentrations of Mg^2+^ (210 μM Murray et al., 1998). Interestingly, the growth defect of an *ezrA* mutant on PAB plates with tetracycline can be suppressed by the addition of Mg^2+^. Tetracycline is a metal-ion chelator (Nelson, 1998) and might reduce the cellular Mg^2+^ concentrations to such levels that growth and cell division are affected in this growth medium. If this is the case then a similar phenotype should be observed with other magnesium chelators. However, neither the addition of EDTA nor phenanthroline, applied at the same molar concentrations as tetracycline (23 μM), had an effect on cell division (not shown).

**Figure 2:**
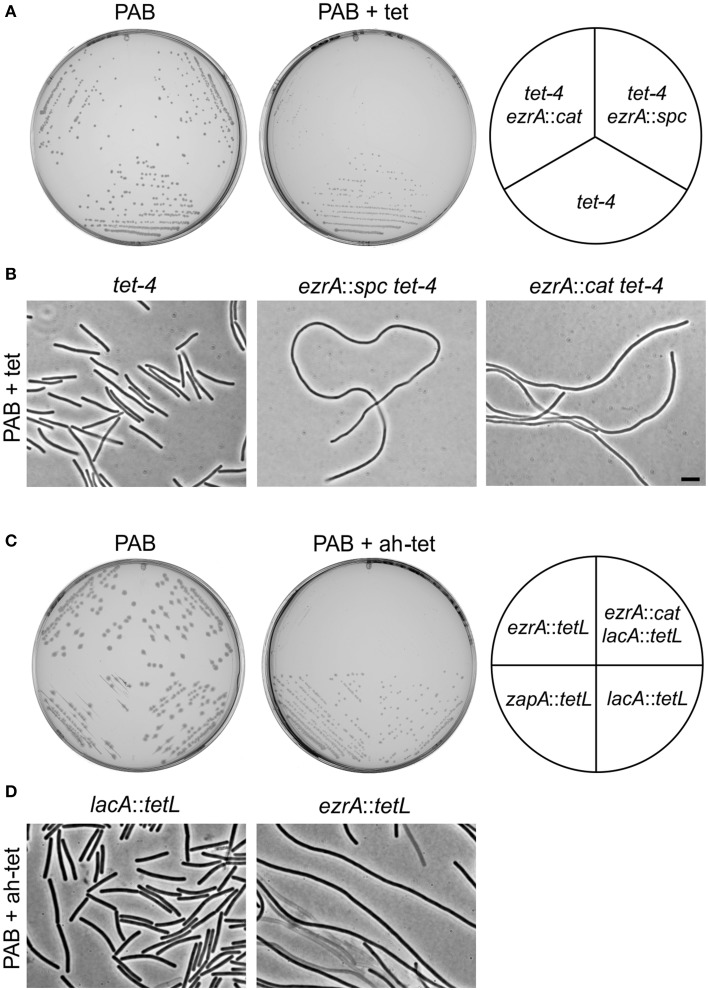
**Tetracycline-induced growth defects of *ezrA* mutants are unrelated to protein translation inhibition. (A)** Growth of strains *tet-4* (PG112), *ezrA*::*cat tet-4* (PG116), *ezrA*::*spc tet-4* (PG113), on PAB plates supplemented with 2 μg/ml tetracycline (tet). **(B)** Phase contrast images of cells taken from the PAB plates in **(A)**. Scale bar 5 μm. **(C)** Effect of anhydrotetracycline. Growth of *ezrA*::*tet* (3362), *ezrA*::*cat lacA*::*tet* (PG100), *zapA-yshB*::*tet* (1356), and *lacA*::*tet* (SG82) strains on PAB plates with or without 0.5 μg/ml anhydrotetracycline (ah-tet). ZapA mutant strain was included as an additional control. **(D)** Phase contrast images of cells taken from the PAB plates in **(C)**.

The *tetL* cassette encodes the TetA transporter which exports tetracycline in a complex with divalent cations such as Mg^2+^ (Krulwich et al., 2001). To see whether the growth phenotype was linked to the presence of the TetA transporter, an alternative tetracycline resistance cassette was used. *tet-4* is a point mutation in the ribosomal protein S10 that reduces the sensitivity of the ribosome for tetracycline (Williams and Smith, 1979; Wei and Bechhofer, 2002). This mutation confers tetracycline resistance without affecting the concentration of the internal Mg^2+^ pool. The *tet-4* mutation provides a lower resistance to tetracycline compared to the *tetL* cassette, therefore strains were grown on PAB plates containing 2 μg/ml tetracycline (Figures 2A,B). Again, introduction of an *ezrA* mutation in the *tet-4* background caused hypersensitivity to tetracycline, indicating that this phenotype is not related to the TetA transporter.

The fact that the *tet-4* mutation is unable to prevent the tetracycline effect suggests that this phenomenon is not associated with the inhibition of protein translation. This was supported by the finding that 0.5 μg/ml anhydrotetracycline, a tetracycline analog that does not bind to the ribosome (Oliva and Chopra, 1992), also affects growth and cell division of *ezrA* mutants (Figures 2C,D).

### Tetracycline does not affect Z-ring formation

**Figure 3:**
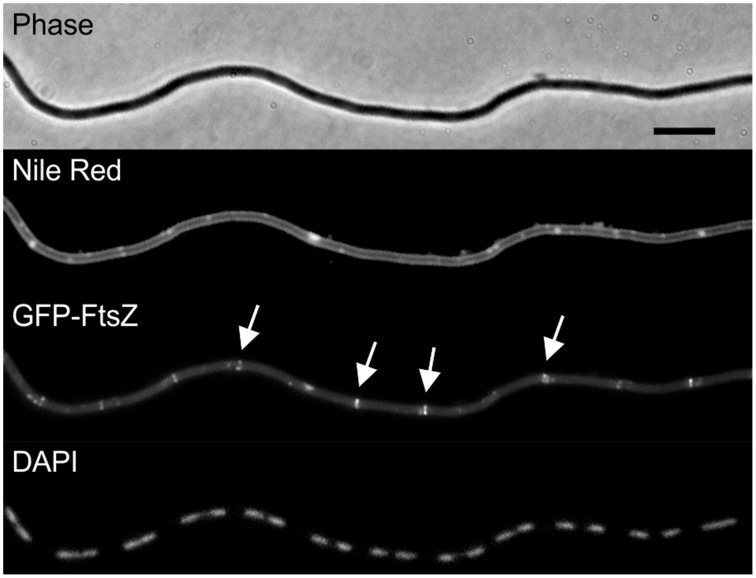
**Tetracycline does not prevent Z-ring formation**. Strain PG209 (*ezrA*::*tet amyE*::*Pxyl-gfp-ftsZ*) was streaked on PAB plates containing 10 μg/ml tetracycline, and 0.5% xylose to induce GFP-FtsZ. Cells were stained with DAPI and Nile Red to visualize nucleoids and the cell membrane, respectively. Arrows highlight some of the Z-rings. Scale bar 5 μm.

The tetracycline-induced filamentation of *ezrA* cells indicates a cell division problem. To examine whether this problem is caused by an inability to form Z-rings, a fluorescent GFP-FtsZ marker was introduced. The resulting strain PG209 (*ezrA*::*tet amyE*::*Pxyl-gfp-ftsZ*) was streaked on PAB plates containing 10 μg/ml tetracycline and 0.5% xylose to induce GFP-FtsZ. Cells were taken from colonies and mounted onto agarose covered microscope slides containing Nile Red and DAPI, to stain the cell membrane and nucleoid, respectively (Figure 3). The filamentous cells were very fragile and many cells lysed. Intact cells showed normal nucleoids and some Z-rings, but the fluorescent membrane stain indicated a clear lack of septation. This suggests that the block in cell division is not caused by a defect in FtsZ assembly, but occurs later in the division process.

### Low FtsL levels in *ezrA* mutants

Kawai and Ogasawara have shown that an *ezrA* mutant is sensitive to reduced FtsL expression levels (Kawai and Ogasawara, 2006). FtsL is unstable and cleaved by the zinc metalloprotease RasP, which is involved in regulated intramembrane proteolysis (RIP) (Heinrich et al., 2008; Wadenpohl and Bramkamp, 2010). This proteolytic degradation plays an important regulatory role in the assembly of the late cell division proteins (Daniel et al., 2006). Possibly, FtsL levels become critically limiting for growth when an *ezrA* mutant is grown on PAB plates in the presence of tetracycline.

**Figure 4:**
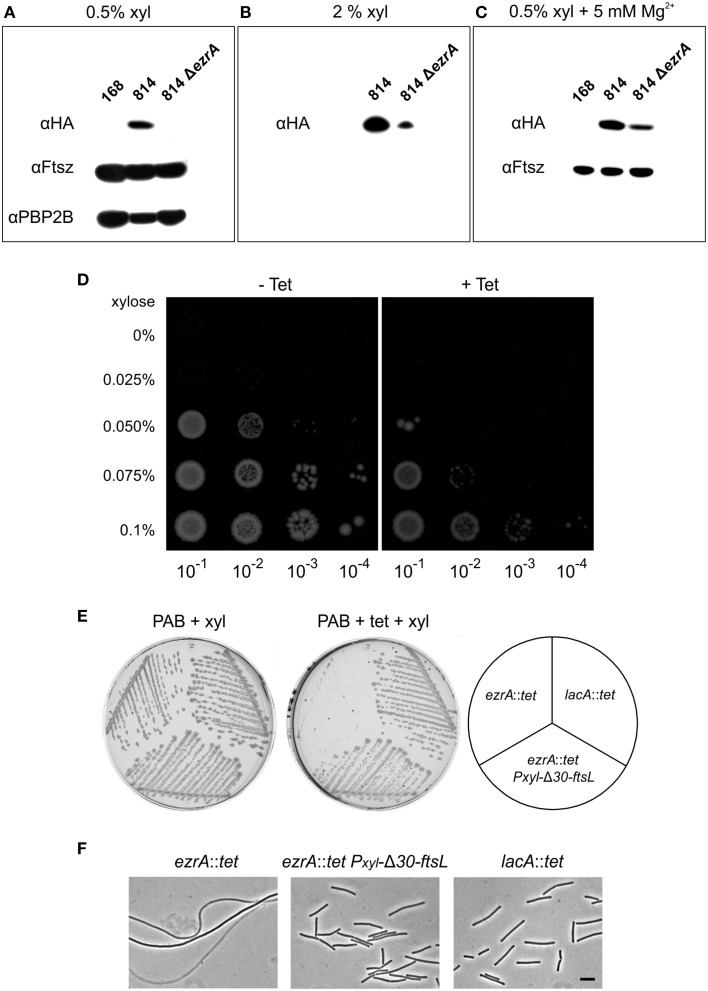
**FtsL overexpression suppresses the tetracycline effect. (A)** Western blot of HA-FtsL, FtsZ, and Pbp2B from total protein extracts of strains 168 (wild type), 814 (Δ*ftsL-Pspac-pbpB, amyE*::*Pxyl-HA-ftsL*), and 814 Δ*ezrA* (PG305) grown at 37°C in PAB medium supplemented with 1 mM IPTG and 0.5% xylose. IPTG was added to express the essential *pbpB* gene downstream of the *ftsl-pbp2B* operon. **(B)** Western blot of HA-FtsL from total protein extracts of strains 814 and 814 Δ*ezrA* (PG305) grown in PAB medium with 1 mM IPTG and 2% xylose. **(C)** Western blot of HA-FtsL and FtsZ from total protein extracts of strains 168, 814, and 814 Δ*ezrA* (PG305) grown at 37°C in PAB medium supplemented with 5 mM MgSO_4_, 20 μg/ml K-aspartate, 1 mM IPTG, and 0.5% xylose. Aspartate was included to circumvent any effect on the inactive downstream *aspB* gene. **(D)** Growth of strain PG742 (Δ*ftsL*-*Pspac*-*pbpB*, *amyE*::*Pxyl*-*HA*-*ftsL*, *lacA*::*tet*) on PAB plates supplemented with 1 mM IPTG, with increasing concentrations of xylose (0.025–0.1%) and with or without 10 μg/ml tetracycline. Serial dilutions of exponentially growing cells were plated and images were taken after overnight incubation at 37°C. **(E)** Growth of *ezrA*::*tet* (3362), *lacA*::*tet* (SG82), and *ezrA*::*tet amyE*::*Pxyl*-Δ*30*-*ftsL* (PG160) strains on PAB plates with 1% xylose, and with or without 10 μg/ml tetracycline, after overnight incubation at 37°C. **(F)** Phase contrast images of cells taken from the plates. Scale bar 5 μm.

We first tested FtsL levels in an *ezrA* background, by using a strain that carries a deletion of the native *ftsL* gene and an ectopically located xylose-inducible HA-tagged *ftsL* fusion (strain 814, Daniel and Errington, 2000). The HA epitope tag enables convenient detection of cellular FtsL levels with Western blotting using sensitive HA-antibodies. Strain 814 was transformed with the *ezrA*::*tet* mutation, resulting in strain PG305. This strain showed an extremely slow growth rate when grown with 0.5% xylose in liquid PAB medium without antibiotics. In fact, no HA-FtsL could be detected under these conditions (Figure 4A). When the xylose concentration was increased to 2%, strain PG305 grew better, although still slower than strain 814, and a weak HA-FtsL band became visible (Figure 4B). Interestingly, strain PG305 showed normal growth with 0.5% xylose when 5 mM Mg^2+^ was added to the medium, although FtsL levels were still not restored to the levels observed in the parental strain 814 (Figure 4C). These data indicate that a deletion of *ezrA* results in reduced FtsL levels, which explains why an *ezrA* mutant is so sensitive for manipulation of the FtsL concentration.

As mentioned above, we observed hypersensitivity to tetracycline only on agar plates. Unfortunately, growth on solid medium hampers homogeneous sampling at specific growth phases. Therefore, we introduced a tetracycline resistance marker into strain 814, obtaining strain PG742 and plated serial dilutions onto PAB plates in the presence of increasing concentrations of xylose, to allow for differential expression of FtsL. As shown in Figure 4D, higher levels of induction were required to allow colony formation in the presence of tetracycline, suggesting that FtsL levels become limiting under those conditions. These data would imply that the tetracycline-induced filamentation of an *ezrA* mutant can be suppressed by increasing FtsL levels in the cell. To test this, a xylose-inducible truncated copy of FtsL (Δ*30-ftsL*) was introduced into an *ezrA*::*tet* mutant. This variant of FtsL was chosen since removal of the first 30 amino acids of FtsL stabilizes the protein (Bramkamp et al., 2006). When the resulting strain PG160 was streaked on PAB plates with tetracycline and 1% xylose, cell division was indeed restored and normal colonies were obtained (Figures 4E,F).

### Screen for novel suppressor mutants

**Figure 5:**
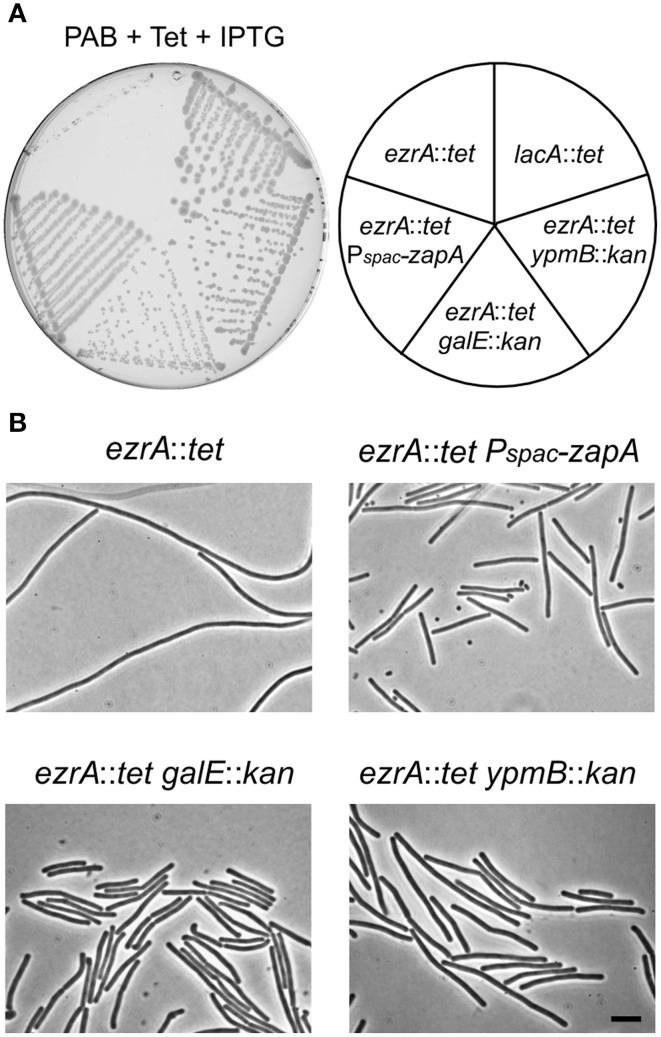
**Suppression of the tetracycline phenotype. (A)** Growth of *ezrA*::*tet* (3362), *ezrA*::*tet aprE*::*Pspac-zapA* (PG273) *ezrA*::*tet galE*::*kan* (PG238), *ezrA*::*tet ypmB*::*kan* (PG239), *lacA::tet* (SG82) strains on a PAB plate with 10 μg/ml tetracycline and 1 mM IPTG for ZapA induction. **(B)** Phase contrast images of cells taken from the PAB plates in **(A)**. Scale bar 5 μm.

The mechanism by which tetracycline causes filamentation of an *ezrA* mutant is unclear. To examine whether other proteins are involved in the tetracycline effect, we screened a transposon library for mutants that would grow normally on PAB plates with tetracycline. Plasmid pMarB, carrying the *mariner* transposon TnYLB-1 (Le Breton et al., 2006), was introduced into strain 3362 (*ezrA*::*tet*), and after transposon mutagenesis approximately 70,000 colonies were screened. Several suppressor mutants that restored colony growth and rescued the division defect were selected. Further analyses showed that one suppressor strain contained a transposon inserted immediately after *zapA*. Two other suppressor mutants harbored transposon insertions into *galE*, which encodes an UDP-galactose epimerase (Estrela et al., 1991; Soldo et al., 2003), and two suppressor mutants contained transposon insertions in the unknown gene *ypmB*. A *galE* and *ypmB* null mutant were made by replacing the complete ORFs with a kanamycin resistance marker (strain PG234 and PG235), and transforming the deletions into strain 3362 (*ezrA*::*tet*). The resulting double mutants grow normally on PAB plates with tetracycline (Figure 5), confirming that the absence of either GalE or YpmB suppresses the tetracycline induced cell division defect.

### *zapA* overexpression suppresses filamentation

**Figure 6:**
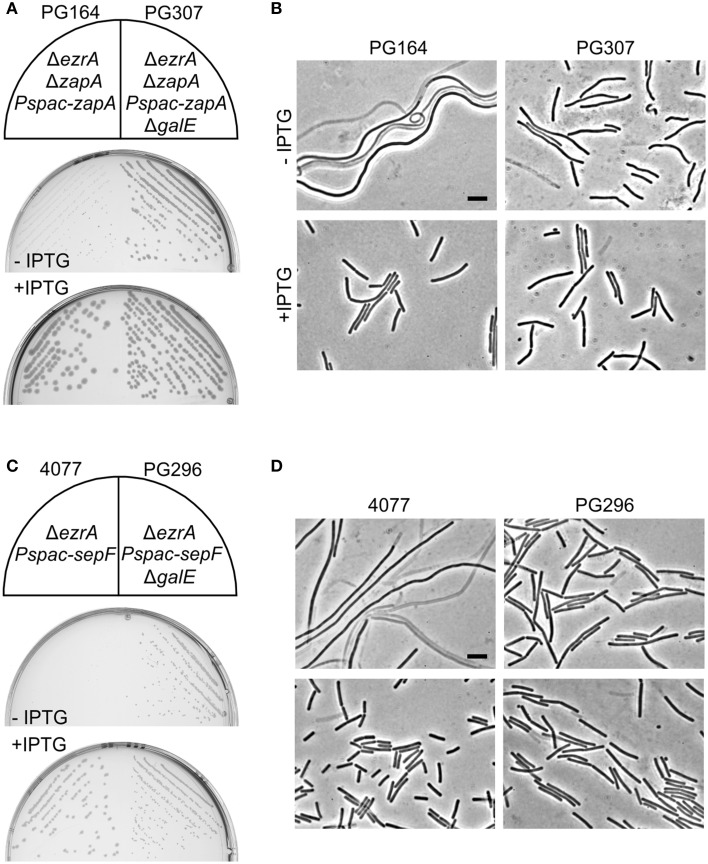
**Absence of GalE restores cell division in *zapA ezrA* and *sepF ezrA* double mutants. (A)** Growth on PAB plates with or without 1 mM IPTG, and **(B)** related phase contrast microscopic images of strains PG164 (*zapA-yshB*::*tet*, *ezrA*::*cat, aprE*::*P_spac_-zapA)* and PG307 (*zapA-yshB*::*tet*, *ezrA*::*cat, aprE*::*P_spac_-zapA, galE*::*kan*). IPTG was used to induce ZapA. **(C)** Growth on nutrient agar plates with 0.5 μg/ml erythromycin, in the presence or absence of 1 mM IPTG, and **(D)** related phase contrast microscopic images of strains 4077 (*ylmBC*::*P_spac_-ylmD-H, ezrA*::*tet)* and PG296 (*ylmBC*::*P_spac_-ylmD-H, ezrA*::*tet, galE*::*kan*). Addition of IPTG induces the expression of *sepF* (= *ylmF*) and of the *ylmDEGH* genes. Scale bar 5 μm.

*zapA* is the upstream gene in the bicistronic *zapA-yshB* operon. Since one of the suppressors contained a transposon insertion between *zapA* and *yshB*, and precisely one nucleotide upstream the start codon of *yshB*, it is possible that a reduced expression of YshB prevents the synthetic filamentous phenotype. To test this, an *ezrA* mutation was introduced into a strain that lacks the complete *zapA-yshB* operon and contains an ectopic copy of only *zapA* driven by the IPTG-inducible *Pspac* promoter. In the absence of IPTG, this strain formed small colonies and highly filamentous cells on PAB plates, even without tetracycline (Figures 6A,B). This is in agreement with a previous study which showed that a *zapA ezrA* double mutant forms very filamentous cells (Gueiros-Filho and Losick, 2002). However, the strain grew normally and showed no filamentation when IPTG was added to induce ZapA expression (Figures 6A,B), even in the presence of tetracycline (not shown). Thus, suppression of the tetracycline phenotype does not require the absence of YshB, since an *ezrA* mutant that overexpresses ZapA and that contains a normal copy of the *zapA-yshB* operon, also grows normally (Figure 5). It is likely that the transposon insertion somehow stabilizes *zapA* mRNA leading to increased ZapA levels in the cell. We therefore tested ZapA levels with Western blot and confirmed that the transposon insertion causes overexpression of ZapA in both 168 and *ezrA* backgrounds (Figure S1).

### Deletion of *galE* restores cell division

GalE is an epimerase that catalyzes the reversible conversion between UDP-galactose and UDP-glucose, as well as between UDP-N-acetylgalactosamine and UDP-N-acetylglucosamine (Krispin and Allmansberger, 1998; Soldo et al., 2003). A *galE* mutant is defective in exopolysaccharide synthesis which results in impaired biofilm formation (Chai et al., 2012). Moreover, the cell wall of a *galE* mutant is devoid of poly(glucose galactosamine 1-P), the so-called minor wall-teichoic acid (Freymond et al., 2006). Teichoic acids are phosphate-rich anionic glycopolymers which constitute a major component of the Gram-positive cell wall. Several physiological roles have been proposed for these polymers, including cation homoeostasis, antibiotic resistance, morphogenesis and cell division (Weidenmaier and Peschel, 2008; Schirner et al., 2009). Interestingly, an *ezrA* mutant is also more sensitive to chloramphenicol and ampicillin as well as to several other cell wall antibiotics (Figures S2, S3). General antibiotic sensitivity is a phenotype that is often observed in cell wall mutants, and in this case might be related to the role of EzrA in shuttling PBP 1 from the lateral wall to the division site (Claessen et al., 2008). Introduction of a *galE* mutation decreased antibiotic sensitivity of an *ezrA* mutant to wild type levels (Figure S3). Importantly, inactivation of *galE* alone showed no increased resistance to antibiotics compared to the wild type strain 168, suggesting that the suppression effect is not due to a general increased protection against antibiotics by means of an altered cell wall composition. Moreover, deletion of the sugar transferases GgaAB, which also impairs minor teichoic acid synthesis (Freymond et al., 2006), did not suppress the tetracycline phenotype.

### Absence of GalE restores cell division in *sepF ezrA* and *zapA ezrA* double mutants

A remaining question is whether the absence of GalE only suppresses the tetracycline effect or whether such mutation actually has a more direct role in cell division. Previously, it was shown that mutations in the lipoteichoic acid biosynthesis pathway reduces the activity of UgtP, thereby stimulating FtsZ polymerization (Weart et al., 2007). Since a *galE* mutation changes the teichoic acid composition of the cell, this mutation might also influence cell division. To test this, the possible mitigating effect of a *galE* deletion on the cell division defect of a *zapA ezrA* double mutant was investigated. A strain lacking *ezrA*, and with *zapA* under control of an IPTG-inducible promoter (strain PG164), forms small colonies and filamentous cells in the absence of IPTG. When the *galE* deletion was introduced into this background, the resulting strain (PG307) grew much better without IPTG and filamentation was strongly reduced (Figures 6A,B).

Since a *galE* deletion restored cell division in the *zapA ezrA* double mutant, we were curious whether such deletion could also restore growth and cell division in the synthetic lethal *sepF ezrA* double mutant. *B. subtilis* strain 4077 contains an *ezrA* deletion and an IPTG-inducible *sepF* operon. This strain can only grow when IPTG is added to the growth medium (Hamoen et al., 2006). However, transformation of the *galE* mutation into this strain resulted in colony formation on PAB plates without IPTG (Figure 6C), and microscopic imaging showed that cell division was restored (Figure 6D). Consistent with this result, it was possible to make a viable *ezrA sepF galE* triple mutant (PG294), although this strain grows slower than the single mutants and shows a high degree of filamentation (Figure S4). Again, the effect of a *galE* deletion is not linked to the lack of minor teichoic acids as the Δ*ggaAB* mutant failed to suppress IPTG dependency of strain 4077 (not shown). Therefore, we must conclude that GalE activity affects the cell division process.

### Deletion of *ypmB* (*tseB*) suppresses the tetracycline effect

Two transposon suppressors were found in *ypmB*, and replacement of *ypmB* by a kanamycin resistant marker suppressed the tetracycline-induced growth inhibition and filamentation (Figure 5). However, *ypmB* is the second gene of a tri-cistronic operon and is preceded by *ypmA* and followed by *aspB*, which is involved in aspartate biosynthesis. To rule out a possible downstream effect, an ectopic IPTG-inducible copy of *ypmB* was introduced into the *ypmB ezrA* double mutant. The resulting strain PG333 forms only filamentous cells on tetracycline containing PAB plates when IPTG is present, indicating that the transposon suppression is due to the absence of a functional *ypmB* gene and not a consequence of downstream effects on *aspB* (Figure S5). Because of its role in the tetracycline sensitivity of an *ezrA* strain, this hypothetical gene was renamed *tseB* (*t*etracycline sensitivity *s*uppressor of *e*zrA).

**Figure 7:**
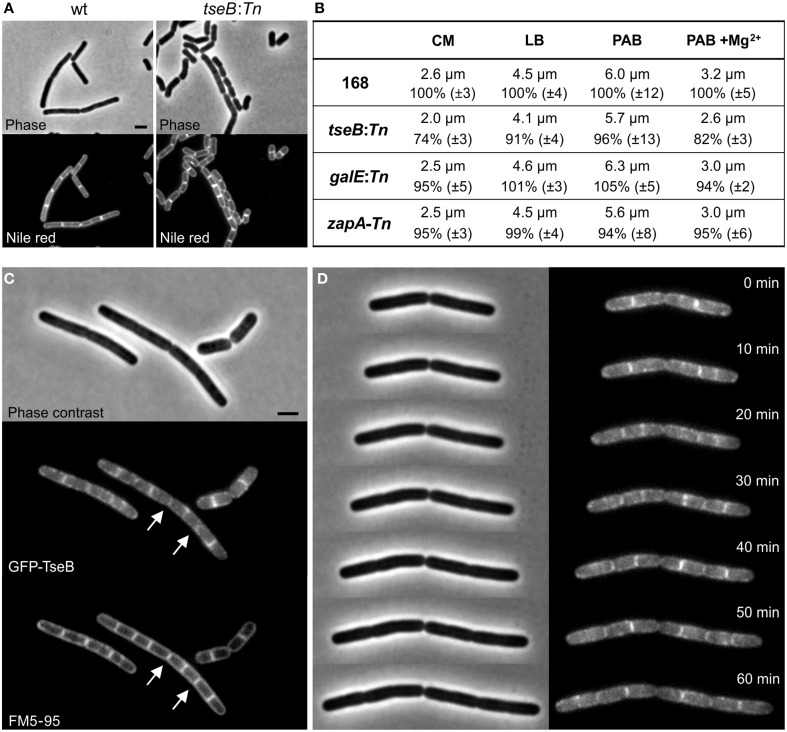
**Phenotype of Δ*tseB* and localization of GFP-TseB. (A)** Phase contrast and membrane stain (Nile red) images of wild type strain 168 and the *tseB* mutant strain PG135 (*tseB*:*TnYLB-1*) grown in competence medium at 37°C. Scale bar 2 μm. **(B)** Cell length measurements of the transposon mutants in different growth media. Strains 168, *tseB*:*TnYLB-1*(PG135), *galE*:*TnYLB-1* (PG143) and *zapA*-*TnYLB1-yshB* (PG140), were grown at 37°C in competence medium (CM), LB, PAB, or PAB supplemented with 5 mM Mg^2+^. Averaged absolute and relative cell lengths are presented below in %, and standard deviations are shown in brackets. One hundred to one hundred and fifty cells were measured in each experiment in triplicate. **(C)** Localization of GFP-TseB. Strain PG718 (*amyE*::*Pxyl-mgfp-tseB*) was grown in competence medium at 30°C with 0.5% xylose to express GFP-TseB. GFP, membrane stain (FM5-95) and phase contrast images were taken during exponential growth. Scale bar 2 μm. Arrows highlight some of the septa in which the GFP signal is absent. **(D)** Time-lapse microscopy experiment showing dynamic localization of GFP-TseB. Strain PG718 (*amyE*::*Pxyl-mgfp-tseB*) was grown at 30°C on a microscope slide made of competence medium supplemented with 0.5% xylose. GFP and phase contrast images were taken every 10 min.

When cell lengths of the different transposon mutants were measured in an otherwise wild type background, the insertion in *tseB* showed the greatest effect and produced significant shorter cells compared to the wild type strain, especially when grown in minimal competence medium (approximately 25% shorter) (Figures 7A,B). Minimal competence medium contains a relative high concentration of Mg^2+^ (6.6 mM), and the addition of magnesium to PAB medium further reduced the average cell length (Figure 7B). The addition of aspartic acid to the growth medium, which might be required if *aspB* was not expressed at sufficient levels, did not have an effect on this phenotype (not shown).

Secondary structure predictions of the 161 amino acid long TseB suggested that the protein has a large extracellular domain attached to the cell membrane by a single N-terminal transmembrane helix (SOSUI software, Hirokawa et al., 1998). To study the localization of TseB, an N-terminal GFP-TseB fusion was constructed using a monomeric variant of GFP. The reporter fusion was inserted into the *amyE* locus of a strain carrying also the wild type copy of *tseB* at the native locus. The GFP-TseB fusion is at least partially functional since it can complement the short cell phenotype of a *tseB* mutation in minimal medium (not shown). The GFP-TseB fusion shows clear membrane localization that is enriched at cell division sites in some cells (Figure 7C). In addition, the GFP signal appears to be almost absent from matured septa (Figure 7C, arrows). Time-lapse microscopy showed this dynamic localization more clearly, and confirmed the disappearance of the protein from septa late in the cell division process, presumably when septation is completed (Figure 7D).

## Discussion

### Hypersensitivity to tetracycline

The finding that a cell division mutant is hypersensitive to antibiotics, and in particular to tetracycline, has not been reported before. It is as yet unclear why tetracycline causes a growth and division defect in an *ezrA* mutant while the tetracycline-resistance marker is present. Our data suggests that FtsL might be destabilized under these growth conditions (Figure 4D). This might lead to a severe division block when combined with an *ezrA* deletion, which has in itself a similar effect (Figures 4A–C). However, our transposon screen revealed that also ZapA overexpression can suppress the tetracycline effect. In contrast to FtsL, ZapA is an early cell division protein and forms links between FtsZ protofilaments promoting Z-ring assembly (Gueiros-Filho and Losick, 2002; Pacheco-Gomez et al., 2013). Possibly, this also promotes the stability of the late divisome components.

The fact the tetracycline hypersensitivity phenotype is only observed on PAB plates and not in liquid medium might have to do with localized depletion of Mg^2+^ ions that exacerbates the effect. Since Mg^2+^ suppresses the tetracycline induced phenotype, we initially assumed that the metal-ion chelating activity of tetracycline was responsible for the cell division defect. However, other metal chelators did not result in filamentation of an *ezrA* mutant. Interestingly, it is not the classical translation-inhibiting activity of tetracycline that is causing cell filamentation, since the *tet-4* ribosomal mutation did not mitigate the tetracycline effect, and anhydrotetracycline also caused filamentation. Tetracycline and anhydrotetracycline are lipophilic compounds that accumulate in the cell membrane, and it has been suggested that the bactericidal activity of anhydrotetracycline is caused by membrane de-energization (Chopra, 1994; Chopra and Roberts, 2001). Reduction of the membrane potential by tetracycline could explain its effects on cell division, since the cell division proteins FtsA and MinD require the membrane potential for membrane localization and function (Strahl and Hamoen, 2010). However, we have been unable to detect a clear reduction in membrane potential (within 15 min) when *B. subtilis* cells were incubated with tetracycline. It has been shown that relative small amounts of tetracycline (1 μg/ml) can increase the membrane fluidity (Vincent et al., 2004). Possibly, this change in membrane fluidity will make certain transmembrane proteins more susceptible to proteolytic degradation, such as FtsL or other late division proteins. Interestingly, divalent cations are known to reduce membrane fluidity (Binder and Zschornig, 2002; Vest et al., 2004), and this might explain why the addition of Mg^2+^ suppresses the tetracycline effect.

### Effect of GalE on cell division

A *galE* deletion suppresses the tetracycline effect and rescues the synthetic lethality of *zapA ezrA* and *sepF ezrA* double mutations. This, together with the fact that the lack of minor teichoic acids itself (Δ*ggaAB* mutant) did not suppress the cell division effect, suggests that GalE plays a more direct role in cell division. We could not observe any division defect in a *galE* mutant, but the *galE* mutation improves the efficiency of division in *ezrA* mutant cells considerably. One possibility is that the absence of *galE* alters the levels of UDP-glucose, since GalE catalyzes the reversible production of UDP-glucose from UDP-galactose. UDP-glucose is the substrate for UgtP, the sugar transferase that is involved in lipoteichoic acid, which also regulates FtsZ assembly (Weart et al., 2007). Therefore, a *galE* deletion might indirectly influence the activity of UgtP. Interestingly, we were unable to test the *ugtP* mutant on PAB plates, since this strain showed strongly impaired growth and morphological defects (bulging) in PAB medium (not shown).

### TseB influences cell division

We have shown that TseB deletion suppresses the tetracycline effect and causes a short cell phenotype under certain growth conditions. The protein is attached to the membrane and contains an extra-cytoplasmic peptidase domain that is found in cell-wall associated regulatory metallopeptidases (Yeats et al., 2004). We hypothesize that the protein might be involved in the proteolytic degradation of extracellular proteins among which FtsL or others that affect the levels of FtsL. However, western blot experiments failed to consistently detect increased amounts of FtsL in a *tseB* mutant (not shown). Nevertheless, the short cell phenotype of a *tseB* mutant and the septal enrichment is compatible with a role in the division process. The protein is conserved within families belonging to the Bacillales and Lactobacillales orders (STRING database, Szklarczyk et al., 2011). Intriguingly, there is a significant co-occurrence in bacterial genomes among TseB, PBP2A and PbpH (STRING database, (Szklarczyk et al., 2011)). These two penicillin-binding proteins are required for cell wall synthesis during elongation (Wei et al., 2003) and were shown to be drivers for MreB dynamics (Dominguez-Escobar et al., 2011; Garner et al., 2011). Possibly, TseB is involved in the switch between septal and lateral cell wall synthesis, which could explain its connection to EzrA.

### Conflict of interest statement

The authors declare that the research was conducted in the absence of any commercial or financial relationships that could be construed as a potential conflict of interest.
