# Supplementary material for: Tetracycline hypersensitivity of an ezrA mutant links GalE and TseB (YpmB) to cell division
Source: Front Microbiol. 2015 Apr 22;6:346. doi: 10.3389/fmicb.2015.00346 (PMC4406074; doi:10.3389/fmicb.2015.00346)
Supplement: Supplementary file 1 [file DataSheet1.PDF]

Supplementary Material

Tetracycline hypersensitivity of an *ezrA* mutant links GalE and TseB (YpmB) to cell division

Pamela Gamba[1,*], Eva Rietkötter[1], Richard A. Daniel[1], Leendert W. Hamoen[1,2,*]

[1] Centre for Bacterial Cell Biology, Institute for Cell and Molecular Biosciences, Newcastle University, Newcastle upon Tyne, UK.
[2] Bacterial Cell Biology, Swammerdam Institute for Life Sciences, University of Amsterdam, Amsterdam, the Netherlands.

*** Correspondence:**

Dr. Leendert W. Hamoen, Bacterial Cell Biology, Swammerdam Institute for Life Sciences (SILS), University of Amsterdam, Science Park 904, 1098 XH, Amsterdam, the Netherlands.
E-mail: l.w.hamoen@uva.nl

Dr. Pamela Gamba, Centre for Bacterial Cell Biology, Institute for Cell and Molecular Biosciences, Newcastle University, Richardson Road, NE2 4AX, Newcastle upon Tyne, UK.
E-mail: pamelagamba@gmail.com

Supplementary Figures

Figure S1. The tetracycline-suppressing transposon insertion downstream *zapA* causes overexpression of ZapA.

Figure S2. Sensitivity to different cell wall antibiotics.

Figure S3. Deletion of *galE* rescues antibiotic sensitivity of an *ezrA* mutant.

Figure S4. *ezrA sepF galE* triple mutant is viable.

Figure S5. Complementation of *ypmB* phenotype.

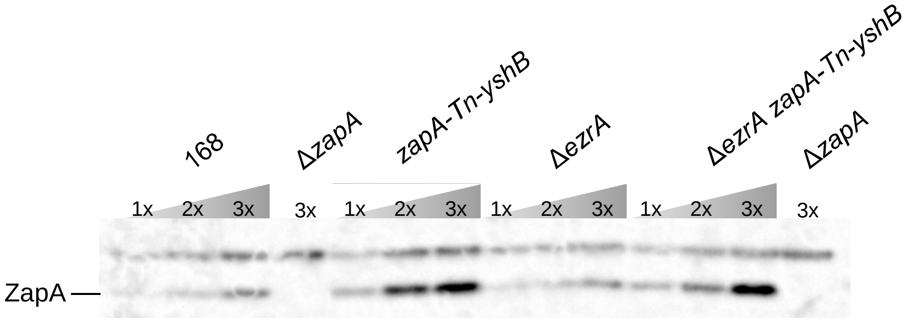

Supplementary figure S1. The tetracycline-suppressing transposon insertion downstream *zapA* causes overexpression of ZapA. Western blot of ZapA from protein extracts of strains 168 (wild type), 1356 (Δ*zapA*), PG140 (*zapA-TnYLB1-yshB*), PG94 (Δ*ezrA*) and PG126 (Δ*ezrA zapA-TnYLB1-yshB*). Strains were grown at 37°C in PAB medium and samples were taken at O.D.600 ~0.3. Protein concentration was measured with Bio-Rad Protein Assay and equal amount of proteins (in 3 linear dilutions) were loaded on a gel. The aspecific band above ZapA band is shown as indication of homogeneous loading and transfer.

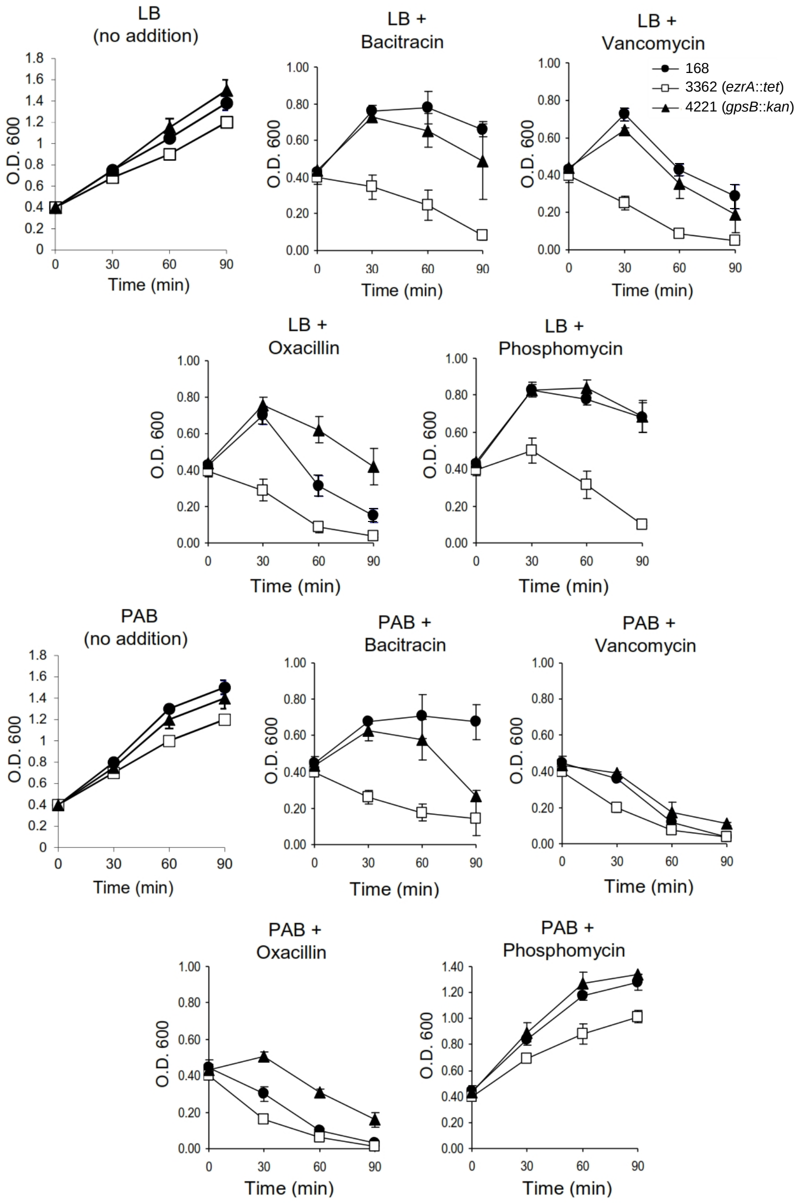

Supplementary figure S2. Sensitivity to different cell wall antibiotics. Strains 168 (●), *ezrA*::*tet* (3362, ☐), and *gpsB*::*kan* (4221, ▲) were grown in LB or PAB at 37°C to an O.D.600 of ~0.8. Cultures were then diluted twofold in warm medium containing 250 µg/ml bacitracin, 1 µg/ml oxacillin, 1 µg/ml vancomycin or 50 µg/ml phosphomycin (final concentrations). Incubation was continued at 37°C and changes in the O.D.600 were recorded for 90 min. The graphs show the average values calculated from at least three independent experiments (error bars indicate the standard error). Cell wall defects have not been described for an *ezrA* mutant so far, although hypothetically they may be compatible with its role in the coordination of cell elongation/cell division cycle together with GpsB (Claessen *et al.*, 2008). Therefore, as a control, we also tested a *gpsB* mutant.

Claessen, D., Emmins, R., Hamoen, L.W., Daniel, R.A., Errington, J., and Edwards, D.H. (2008). Control of the cell elongation-division cycle by shuttling of PBP1 protein in *Bacillus subtilis*. *Mol Microbiol* 68**,** 1029-1046.

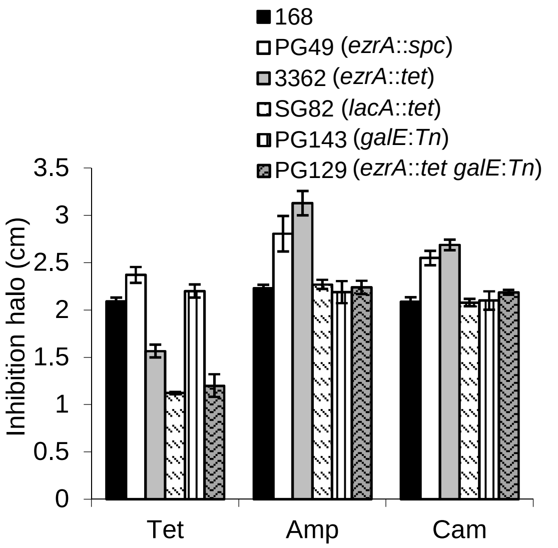

Supplementary figure S3. Deletion of *galE* rescues antibiotic sensitivity of an *ezrA* mutant. Sensitivity to tetracycline (tet), ampicillin (amp) and chloramphenicol (cam) are indicated by the diameter of growth inhibition halos. The average diameter was calculated from at least four independent experiments. Sensitivity of strains 168, *ezrA*::*spc* (PG49), *ezrA*::*tet* (3362), *lacA*::*tet* (SG82) *galE*:TnYLB-1 (PG143) and *ezrA*::*tet galE*:TnYLB-1 (PG129) are shown from left to right. Standard deviation is indicated by error bars. Note that 3362 and SG82 contain the tetracycline resistant gene *tetL*. *spc* depicts the spectinomycin resistance gene, and the transposon contains a kanamycin resistant gene.

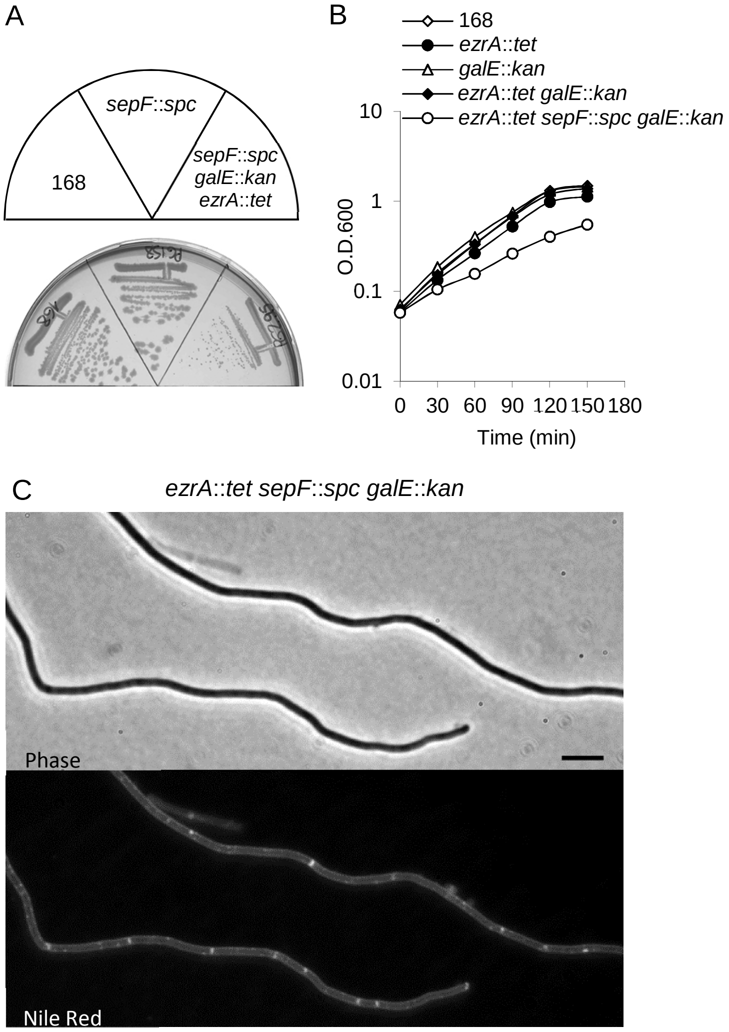

Supplementary figure S4. *ezrA sepF galE* triple mutant is viable. (A) Growth of strains 168, *sepF*::*spc* (PG158) and triple mutant *ezrA*::*tet sepF*::*spc galE*::*kan* (PG294) on a nutrient agar plate after incubation overnight at 37°C. (B) Growth curves of strains 168 (◇), *ezrA*::*tet* (3362, ●), *galE*::*kan* (PG234, △), *ezrA*::*tet*, *galE*::*kan* (PG238, ◆) and triple mutant *ezrA*::*tet sepF*::*spc galE*::*kan* (PG294, ○) in liquid PAB at 37°C. (C) Phase contrast and Nile red images of PG294 cells grown in liquid PAB at 37°C. Samples were taken at O.D.600 ~0.4 and stained with the membrane dye Nile Red. Scale bar 5 µm.

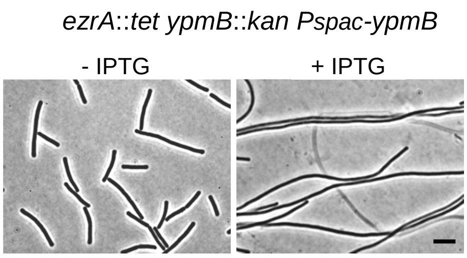

Supplementary figure S5. Complementation of *ypmB* phenotype. Strain PG333 (*ezrA*::*tet ypmB::kan Pspac-ypmB)* was grown on PAB plates with tetracycline, in the presence or absence of 1 mM IPTG. Images were taken after incubation overnight at 37°C. Scale bar 5 µM.
